# An unusual complication following radiological percutaneous gastrostomy

**DOI:** 10.1186/1755-7682-1-15

**Published:** 2008-08-12

**Authors:** Tonny Veenith, Manasi Bhagwat, Andrew Bailey

**Affiliations:** 1 Specialist Registrar, Department of Neurocritical Care Medicine, Addenbrookes Hospital, Cambridge, UK; 2 Consultant in Anaesthesia, Addenbrookes Hospital Cambridge, UK

## Abstract

**Introduction:**

Intestinal malrotation is a condition, which is predominantly recognised in childhood. Because of the relative rarity, there is a possibility that it can be missed in the routine clinical care of adults. This case highlights the need for a high index of suspicion for malrotation when things go wrong in routine procedures. This can be the reason for catastrophic sepsis in patients who undergo minimally invasive procedures.

**Case presentation:**

We present a patient with a malignant lesion of the tongue who went for elective placement of feeding tube who suffered unexpected complication as a result of malrotated large bowel.

**Conclusion:**

Malrotation of the intestine can make a relatively straightforward procedure fraught with complications. Clinicians should have a high index of suspicion about malrotation when performing procedures like percutaneous gastrostomy and radiologically guided entrostomy. If there is an index of suspicion they should be screened prior to the procedure.

## Introduction

Malrotation of the intestine is a well defined aberrancy of development in which the intestines are abnormally placed in the peritoneal cavity and can involve the large and small intestine [1]. We present a patient with an anatomical abnormality of the caecum, which resulted in iatrogenic perforation of the caecum secondary to percutaneous gastrostomy.

## Case presentation

A 57 year old gentleman presented to the hospital with a one month history of dysphagia, neck swelling, ulcer in the tongue and weight loss. He had lost approximately 12 kilograms weight over the previous 2 months. On further investigation he was found to have a squamous cell carcinoma of the tongue with lymph node metastasis. Planned treatment consisted of radical neck dissection with resection of the primary tumour and postoperative radiation therapy. Because of his poor nutritional status it was decided to insert a radiological gastrostomy (RIG) for feeding prior to the procedure.

He had a RIG, performed by interventional radiologist under fluoroscopic guidance with insufflation of the stomach with a nasogastric tube and using a push technique to insert the gastrostomy tube. This procedure was seemingly uneventful and patient was send back to the ward. Six hours later he developed pyrexia, hypotension and clinical evidence of peritonitis, and was transferred to intensive care unit for resuscitation and further treatment. An urgent erect chest x-ray showed free gas under his diaphragm. At subsequent laprotomy a perforated gangrenous malrotated caecum was found in the left hypochondrium overlying the stomach. This was thought to be a complication of the radiological percutaneous gastrostomy. An extended right hemicolectomy with end to end anastomosis was performed. A jejunostomy was inserted for feeding at laprotomy.

His stay in intensive care unit was complicated by right lower lobe pneumonia, but he was fit for discharge to the ward after one week. One month later he underwent his radical neck dissection, after which he had another overnight stay in intensive care unit, and he was subsequently discharged home without any further problems.

## Discussion

The normal position and orientation of the small and large intestine in the abdominal cavity are a result of rotation of the midgut beginning during the fifth week of gestation and continuing into the postnatal period. Any problems with this rotation are termed as malrotation of the intestine, and this can affect small or large intestine. During embryological development the midgut develops further into the jejunum, ileum, caecum, appendix and part of the large intestine. Some authors however believe that this may not be the case and all parts of midgut develop in a desynchronised fashion [2-4].

Estimated incidence of malrotation vary and occurs in up to 0.3% of live births and has been found in up to 1% of autopsies [5-7]. In adults this malrotaion is usually found incidentally as a part of routine scans or unrelated investigations [4]. However in this patient it was only diagnosed after an unexpected complication of a minimally invasive procedure. We believe that his has not been described in the literature before. Other well described causes of caecal perforation are trauma (e.g. following colonoscopy), inflammatory bowel disease, malignancy and diverticulitis.

In a patient with undiagnosed malrotation, the only clinical history that can suggest the condition is chronic abdominal pain. Diagnosis invariably relies on radiological investigations although insufficient evidence precludes routine use. The investigation of choice remains the gastro intestinal series of x-rays and contrast enhanced CT scan of the abdomen [8].

### RIG versus PEG in upper gastrointestinal malignancy

Optimal feeding is essential for patients who are malnourished, requiring major surgery and chemotherapy afterwards, and enteral feeding is nearly always preferred over parenteral feeding [9]. According to the single centre series and metaanalysis by Wollman et. al surgical gastrostomy and percutaneous endoscopic gastrostomy is associated with more complications than RIG and higher success rates of insertion. [10,11]. The insertion of the RIG was also associated with shorter procedure duration, and less usage of sedating agents such as midazolam [10]. RIG was performed even when there is a contraindication to PEG with good results [12]. In this particular patient the insertion of the PEG would possibly have been associated with the same outcome, as the stomach was insufflated with air, which brought it into apposition with the anterior abdominal wall and displaced the colon downwards, which would have been the technique used for PEG as well. But if the anomaly in question is an interposition of the colon it is associated with a displacement of the colon on insufflation of air [13].

## Conclusion

In this patient, the caecal perforation during the radiological gastrostomy was a result of previously undiagnosed malrotation of the caecum. This condition is diagnosed with relative rarity in adult population, however all physicians should be aware of the malrotation and a high index of suspicion should be maintained, particularly when unexpected complication arise after percutaneous gastrointestinal procedure, and radiological examination prior to RIG should be performed if there is any index of suspicion.

## Abbreviations

RIG: Radiological gastrostomy; PEG: Percutaneous gastrostomy.

## Conflicting interests

The authors declare that they have no competing interests.

## Authors' contributions

All authors were involved in writing this manuscript.
